# Upregulation of CIRP by its agonist prevents the development of heart failure in myocardial infarction rats

**DOI:** 10.1186/s12872-024-03852-9

**Published:** 2024-03-27

**Authors:** Jingjing Zhang, Tao Liu, Yanzhao Wei, Jianye Peng, Gaofeng Zeng, Peng Zhong

**Affiliations:** 1 Department of Cardiology Research Institute, Renmin Hospital of Wuhan University, 238 Jiefang Road, Wuhan, Hubei 430060 China; 2 Cardiovascular Research Institute of Wuhan University, Wuhan, 430060 China; 3 Hubei Key Laboratory of Cardiology, Wuhan, 430060 China; 4 The Second Affiliated Hospital, Department of Cardiovascular Medicine, Hengyang Medical School, University of South China, Hengyang, China; 5 The Second Affiliated Hospital, Key laboratory of Heart Failure Prevention & Treatment of Hengyang, Hengyang Medical School, University of South China, Hengyang, 421001 China; 6 Clinical Medicine Research Center of Arteriosclerotic Disease of Hunan Province, University of South China, Hengyang, 421001 China

**Keywords:** CIRP, Myocardial infarction, zr17-2, Inflammation, Nrf2

## Abstract

**Background:**

Downregulated expression of cold-inducible RNA binding protein (CIRP), a stress-response protein, has been demonstrated in the hearts of patients with heart failure (HF). However, whether CIRP plays a critical role in the pathogenesis of HF remains unknown. Zr17-2 is a recently identified CIRP agonist, which can enhance the expression of CIRP in hearts. Herein, we evaluated the effects of zr17-2 on the development of HF in a rat model of myocardial infarction (MI).

**Methods:**

Male SD rats were pretreated with CIRP agonist zr17-2 or vehicle saline for 6 consecutive days, followed by MI induction. 1-week post-MI, cardiac function, and structural and molecular changes were determined by echocardiography and molecular biology methods.

**Results:**

Excitingly, we found that pretreatment with zr17-2 significantly attenuated MI-induced cardiac dysfunction and dilation, coupled with reduced infarction size and cardiac remodeling. In addition, increased inflammatory response in the peri-infarcted heart including macrophage infiltration and the expression of inflammatory genes were all significantly decreased by zr17-2 pretreatment, suggesting an anti-inflammatory effect of zr17-2. Moreover, zr17-2 pretreatment also upregulated the antioxidant genes (e.g. NQO-1, Nrf2, and HO-1) level in the hearts. In isolated cultured cardiomyocytes, pretreatment with zr17-2 markedly attenuated cell injury and apoptosis induced by oxidative injury, along with elevation of Nrf2-related antioxidant genes and CIRP. However, silencing CIRP abolished zr17-2’s antioxidant effects against oxidative injury, confirming that zr17-2’s role is dependent on CIRP.

**Conclusion:**

Collectively, our study suggests CIRP plays a crucial role in the development of HF and a beneficial effect of CIRP agonist in preventing MI-induced HF, possibly via anti-inflammatory and anti-oxidant pathways.

**Supplementary Information:**

The online version contains supplementary material available at 10.1186/s12872-024-03852-9.

## Introduction

 Over two decades ago cold-induced RNA-binding protein (CIRP) was discovered during the investigation of the mechanisms by which mammals adapt to cold stress. Previous studies have demonstrated that as a general stress-responsive protein, the expression of CIRP can be regulated by various stress conditions, including heat stress, hypoxia, UV irradiation, H2O2, and glucose starvation [1, 2]. Under these stressful conditions, CIRP can migrate to the cytoplasm from the nucleus and subsequently regulates mRNA stability via binding to the 3’-UTR of target mRNA. Currently, multiple cellular processes are affected by CIRP, such as telomere maintenance, circadian rhythm regulation, cell proliferation, and cell survival [1, 2].

Heart failure (HF) is a multifactorial disease and often occurs in the end stage of many cardiovascular diseases. Myocardial infarction (MI) is a leading cause of HF with reduced ejection fraction [3]. After a heart attack, the immune system helps repair ischemic damage and restores tissue integrity. However, excessive inflammation can promote adverse cardiac remodeling and the genesis of HF [4]. Preconditioning with interventions such as ischemia has shown beneficial effects in reducing infarct size and pathological remodeling in acute myocardial infarction [5]. Pharmacological preconditioning is also a practical approach to protecting the heart under stressful conditions [6]. The cardioprotective mechanisms of these preconditioning interventions/agents are associated with the activation or blockade of underlying signaling pathways that contributed to disease progression [6]. The prognosis of patients with HF is still not ideal despite great improvements in the management of HF. Thus, a new treatment strategy is needed.

Recent studies suggest a beneficial effect of CIRP on the heart. For instance, CIRP was reported to prevent the heart from apoptosis and dysfunction during ex-vivo extended hypothermic heart preservation, as more severe cell apoptosis and a worse cardiac function were found in CIRP knockout rat hearts, whereas CIRP transgenic rat hearts showed the less apoptotic rate of cardiomyocytes and a better cardiac function [7]. Mechanistic studies showed that CIRP could increase the expression of ubiquinone biosynthesis protein COQ9 at the post-transcriptional level, which could further enhance the biosynthesis of ubiquinone COQ10, thus promoting the production of ATP and protect cells against injury induced by oxidative stress [7]. In addition, treatment with a CIRP agonist zr17-2 has also been shown to extend heart preservation coupled with increased COQ_10_, ATP levels, and scavenging of reactive oxygen species via elevating the protein level of CIRP [7]. Collectively, these results suggest a beneficial role of CIRP in the heart and pharmacological activation of CIRP by zr17-2 may be a promising strategy in ameliorating heart damage under stress conditions. Moreover, our previous study also found that myocardial CIRP was downregulated in HF patients and post-infarction animals, suggesting a possible role of CIRP in HF [8]. Additionally, the knockdown of CIRP has been shown to exacerbate H_2_O_2_-induced cell apoptosis and cell death in cardiomyocytes [8]. Taken together, these results suggest a potential role of CIRP in the pathogenesis of HF, and the downregulation of CIRP might result in cardiomyocyte apoptosis.

However, currently, there is limited literature that investigates the role of CIRP in HF in vivo, and whether the activation of CIRP could prevent the development of HF is still unknown. Zr17-2 is a recently identified CIRP agonist, which is selected based on the properties of compounds that can bind and modulate the activity of CIRP by using high-throughput virtual screening and cell-based western blot assay [9]. Zr17-2 has been demonstrated to enhance the expression of CIRP in various organs including the heart [9]. In this study, we evaluated the effects of the CIRP agonist on the development of HF in a rat model of MI.

## Methods

### Reagents

Zr17-2 was purchased from AOBIOUS INC (Cat No: AOB33334, USA). Fetal bovine serum (FBS), DMEM medium, streptomycin, and penicillin were obtained from GIBICO (USA) for cell culture.

### Ethical approval

Male Sprague-Dawley (SD) rats (6 weeks, weighing from 180 to 220g) were purchased from HFK Biotechnology Company (Beijing, China). All the animal experiment protocols were approved by the Animal Care and Use Committee of Renmin Hospital of Wuhan University and in accordance with ARRIVE gudelines. During the experiment, rats were housed in a temperature-regulated room where the ambient temperature was 22°C and the humidity was 50%. Rats were provided with food and water ad libitum in a 12-h:12-h light/dark cycle. The rats were placed in a bell jar at the end of the experiment, and they were deeply anesthetized with isoflurane for euthanasia.

### Myocardial Infarction model

Rats were randomized to sham, MI+saline, and MI+zr17-2 groups. Rats were anesthetized with pentobarbital sodium (40mg/kg, IP) and ventilated by endotracheal intubation using a Zoovent ventilator. MI was induced by ligating the left anterior descending coronary artery (LAD) as previously described [10]. Successful occlusion of the artery was confirmed by the ST-segment elevation in the electrocardiogram (ECG). The MI+zr17-2 group was treated with zr17-2 (20nmol/kg, i.p.) once every other day 3 times before the operation. The sham group underwent the same procedure as MI-operated rats except for the ligation of LAD.

### Echocardiography

Echocardiography was performed using a Vevo2100 imaging system (Visual Sonics Inc., Toronto, Canada). Rats were anesthetized with pentobarbital sodium (40mg/kg), and transthoracic echocardiography was performed by an experienced operator to assess cardiac function. The left ventricular (LV) ejection fraction (LVEF), LV internal diameters in end-diastole (LVIDd), LV fractional shortening (LVFS), LV end-diastolic volume (LVEDV), LV end-systolic volume(LVESV), LV internal diameter at end-systole (LVIDs) and interventricular septum diameter at end-diastole (IVSD) were detected by M-mode tracing system.

### Histological analysis

Rats were euthanized and hearts were isolated and immersed in buffered formalin for 24 hours.LV tissues were cut into a series of 5-μm-thick slices and stained with hematoxylin-eosin (H&E) and Sirius red to evaluate collagen deposition, or stained with CD68 antibody followed by FITC conjugated-secondary antibody to evaluate macrophage infiltration. Nuclei were counterstained with DAPI and sections were examined with a fluorescent microscope. The percentage of LV circumference was measured to evaluate infarction size at 7 days post-infarction.

### Quantitative real-time PCR

Total RNA was obtained using the Trizol reagent. 5 μg RNA was used to synthesize complementary DNA. qRT-PCR was performed using an Applied Biosystems 7500 Fast Dx Real-time PCR instrument (Thermo Fisher). The primer sequences were shown in Table 1. β-action was used as an internal reference and the fold alterations of each target mRNA level relative to β-actin under different conditions were detected based on the threshold cycle (CT) as r=2^-Δ(ΔCT)^, where ΔCT=CT (target)-CT (β-action) and Δ(ΔCT) = ΔCT (experimental)- ΔCT (control).

**Table 1:** The primer sequences for qRT-PCR

Gene	species	Forward primer	Reverse primer
β-actin	Rat	GACGTTGACATCCGTAAAGACC	CTAGGAGCCAGGGCAGTAATCT
IL-6	Rat	GAGTTGTCAATGGCAATTC	ACTCCAGAAGACCAGAGCAG
IL-1β	Rat	CACCTCTCAAGCAGAGCACAG	GGGTTCCATGGTGAAGTCAAC
VCAM-1	Rat	TCAACTGCACGGTCCCTAAT	TGTGCCAATTTCCTCCCTTA
ICAM-1	Rat	AGATCATACGGGTTTGGGCTTC	TATGACTCGTGAAAGAAATCAGCTC

### Enzyme-linked immunosorbent assay (ELISA)

Serum interleukin-6 (IL-6) level was detected using rat commercially available ELISA assay kit (R&D SYSTEM), relying on the product description.

### Cell culture and treatment

H9C2 cells (obtained from the Shanghai Institute of Biochemistry and Cell Biology, Shanghai, China) were cultured using DMEM containing high glucose (4.5 g/L) ,10% heat-inactivated FBS, penicillin(100 units/ml), and streptomycin(100 µg/ml) at 37℃in a 5% CO2-humidified incubator. Cells were pretreated with zr17-2 (10µmol/L) for 2 days before the stimulation with H_2_O_2_(600µM) for 24 h. Subsequently, cells were subjected to the assay of CCK8 and western blot study.

### siRNA transfection

All chemical CIRP-targeting siRNA (siCIRP) and nonsense siRNA (negative control siRNA, siCtrl), described in this study were synthesized by Genscript (Shanghai, China). After cells were plated, siRNA was mixed with lipofectamine RNAiMAX (catalog number:13,778,075, Invitrogen) and then added into cell culture for 48 h according to the manufacturer’s instructions. The final concentration for the nonsense siRNA or CIRP siRNA was 10 nM. The silencing efficiency of the siRNA was verified by the protein level of the targets.

### Western blot analysis

Total proteins were extracted from the left ventricular tissues. The protein concentration was determined using the Pierce BCA protein Assay kit (Pierce) and 50 µg protein was collected for SDS-PAGE (Invitrogen) and transferred to a polyvinylidene fluoride membrane (Millipore). Proteins were then incubated with several primary antibodies [NQO-1 (ab2346), IL-1β (ab9722), CIRP (ab106230), collagen III (ab7778), and collagen 1(ab6308) obtained from Abcam; ICAM-1 (sc-107), Bax (sc-7480), VCAM-1(sc-13,160), Bcl2(sc-7382), Nrf2 (sc-81,342), and HO-1 (sc-136,960), GAPDH (sc-47,724), obtained from Santa Cruz; cleaved-caspase 3 (#9661) and TGF-β1 (#3711), purchased from Cell Signaling; collagen I (PA1-26204), purchased from Invitrogen] overnight at 4℃ and then incubated with HRP-conjugated secondary antibodies at room temperature for another 2-hour and the blots were visualized with ECL (Bio-Rad) reagent. The protein expression was normalized to GAPDH.

### Statistical analysis

Data are represented as the mean ± S.D. Data were analyzed by one-way ANOVA, followed by Tukey’s Multiple Comparison Test. All statistical analyses were performed in GraphPad Pro 5.0. Statistical significance was set at *P* < 0.05 (*p < 0.05, **p < 0.001, ***p < 0.001 and ****p < 0.0001).

## Results

### zr17-2 pretreatment attenuated cardiac dysfunction and dilatation in MI rats in vivo

**Fig. 1:**
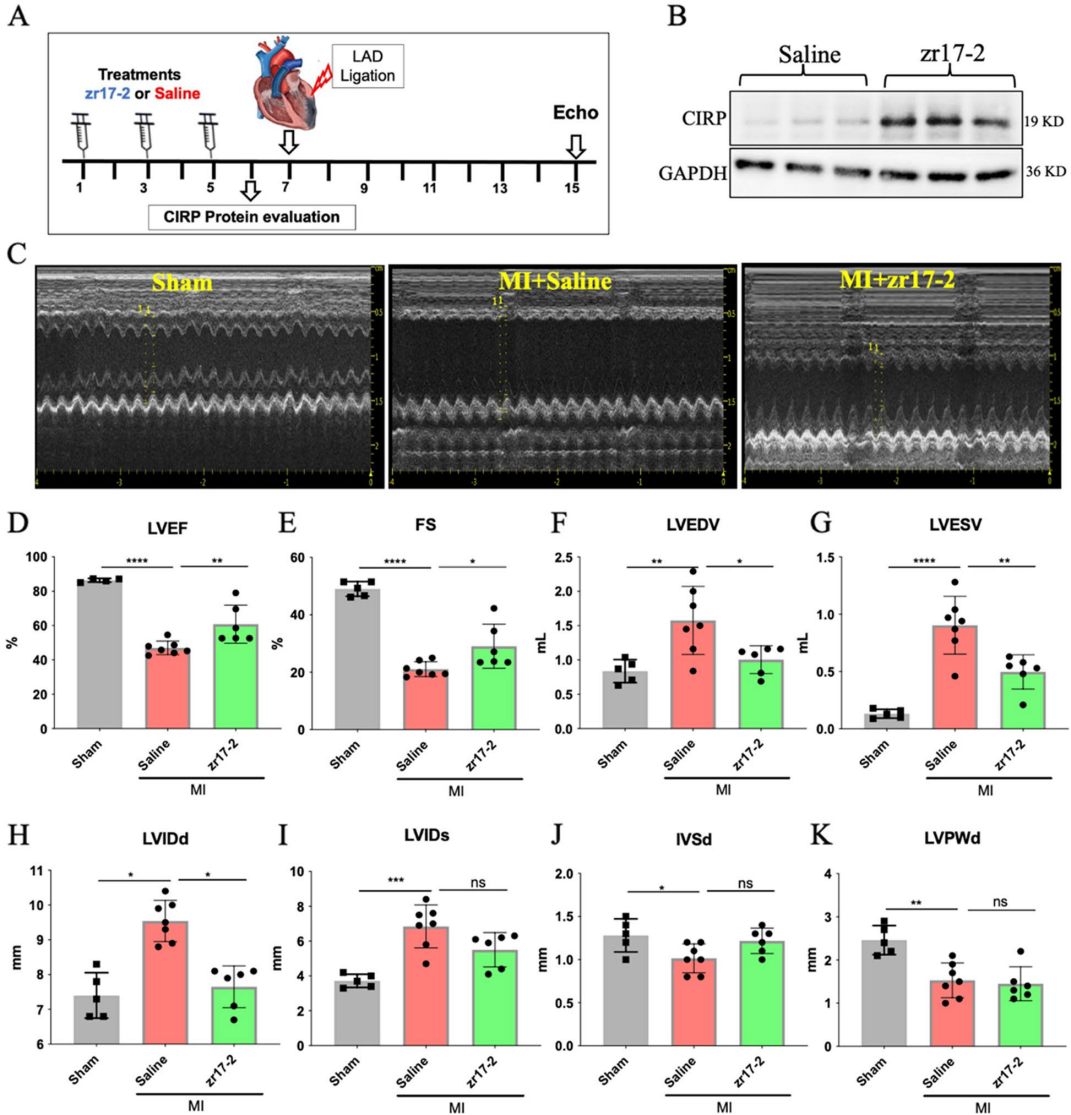
The effects of zr17-2 pretreatment on cardiac function and structure in MI rats. **A** The schematic image depicting the timeline of MI induction and treatment with CIRP agonist zr17-2. **B** Western blot analysis of the CIRP level in the hearts of the rats pretreated with zr17-2 before the induction of MI, GAPDH was used as an internal control. **C** Representative M-mode echocardiograms at the end of the experiment. **D** LVEF; **E** FS; **F** LVEDV; **G** LVESV; **H** LVIDd; **I** LVIDs; **J** IVSd; **K** LVPWd; MI, myocardial infarction; LVEF, left ventricular ejection fraction; FS: left ventricle fractional shortening; LVEDV: left ventricle end-diastolic volume; LVESV: left ventricle end-systolic volume; LVIDd: left ventricle internal diameter at end-diastole; LVIDs: left ventricle internal diameter at end-systole; IVSd: interventricular septum diameter at end-diastole; Data are expressed as the means ± SD; *n* = 4–6 in each group. *p < 0.5, ** *p* < 0.01, ***p < 0.001 and ****p < 0.0001

To determine whether elevation of CIRP would have any beneficial effects in the MI-induced heart failure rat model, we used zr17-2 as a research tool to test the possible effect of CIRP elevation on the development of MI-induced heart failure. Before the induction of the MI rat model, we firstly pre-treated the rats with zr17-2 or saline by peritoneal injection every two days for 3 times, followed by the induction of MI, aiming at elevating cardiac CIRP expression before the MI induction (Fig. 1A). The dose of zr17-2 was chosen based on the previous work [9]. Western blot analysis confirmed that peritoneal injection of zr17-2 significantly upregulated the protein level of CIRP in the heart (Fig. 1B). Echocardiography analysis of 1week post-MI rats showed that MI rats have significantly left ventricular dysfunction and LV cavitary dilation as evidenced by the decrease in LVEF (47 ± 3.93% vs. 86.35 ± 1.05%) and FS (21.07 ± 2.58% vs. 49.02 ± 2.542) and, the increase in LVEDV (1.57 ± 0.49 ml vs. 0.83 ± 0.16 ml), LVESV (0.90 ± 0.25 ml vs. 0.13 ± 0.03 ml), and LVIDd (9.54 ± 0.59 mm vs. 7.4 ± 0.65 mm) in the MI + saline rats compared with that in sham rats (Fig. 1C, D and E, F, G and H). Interestingly, pretreatment with zr17-2 significantly improved cardiac dysfunction and dilation compared with that in the saline-treated group (LVEF:60.84 ± 11.08% vs. 47 ± 3.93%; FS:29.07 ± 7.69% vs. 21.07 ± 2.58%; LVEDV:1.0 ± 0.20 ml vs. 1.57 ± 0.49 ml; LVESV:0.49 ± 0.14 ml vs. 0.90 ± 0.25 ml; LVIDd:7.65 ± 0.59 mm vs. 9.54 ± 0.59 mm) (Fig. 1C-H). However, no significant difference were identified in the LVIDs, IVSd and LVPWd between the the MI + saline group and the MI + zr17-2 group (Fig. 1I-K). Taken together, these results indicated that upregulation of CIRP by its agonist zr17-2 could attenuate cardiac dysfunction and dilation in MI-induced heart failure rats.

### zr17-2 pretreatment reduced cardiac infarction size and improved cardiac remodeling in MI rats in vivo

**Fig. 2:**
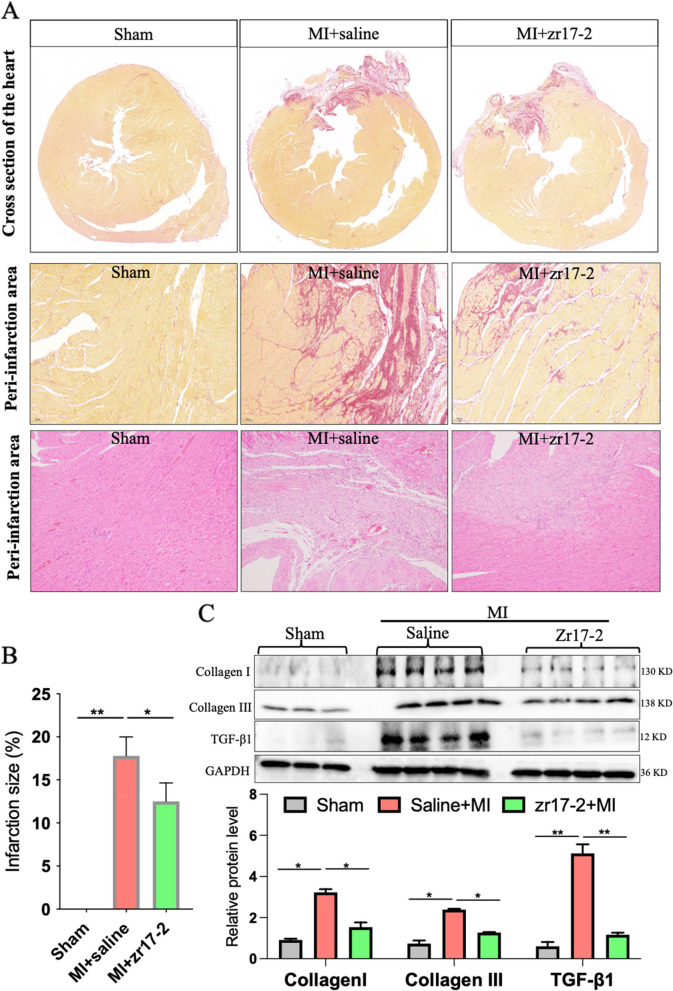
The effects of zr17-2 pretreatment on infarction size and cardiac remodeling in MI rats. **A** Representative Sirius red-stained and H&E-stained images of the heart 7 days post-MI. **B** Quantitative Infarction size based on Sirius red staining sections between different groups. **C** Western blot analysis of the fibrotic markers (collagen I, Collagen III, and TGF-β1) in the peri-infarction region of the heart. Data are expressed as the means ± SD. *n* = 4–6 in each group. * *p* < 0.5, ** *p* < 0.01, *** *p* < 0.001 and ****p < 0.0001

In examining the gross morphology of hearts harvested from the different groups, we found that the ligation of LAD successfully induced infarction (as evidenced by the scar area) in both the MI + saline group and MI + zr17-2 group. However, the degree of infarction size (quantification of the scar area) in the rats from the MI + saline group was significantly higher than that in the rats from the MI + zr17-2 group (Fig. 2A and B). In addition, microscopic evaluation of the peri-infarction area stained with Sirius red showed that there was more connective tissue in the MI + saline group than that in the MI + zr17-2 group (Fig. 2A). Consistently, western blot analysis of the heart from the peri-infarction area also showed more fibrotic markers expression in the MI + saline group and less fibrosis in the MI + zr17-2 group (Fig. 2C). Taken together, these results suggest that pretreatment with zr17-2 reduced cardiac infarction and improved cardiac remodeling in the MI rats.

### zr17-2 pretreatment reduced cardiac inflammatory response in MI rats in vivo

**Fig. 3:**
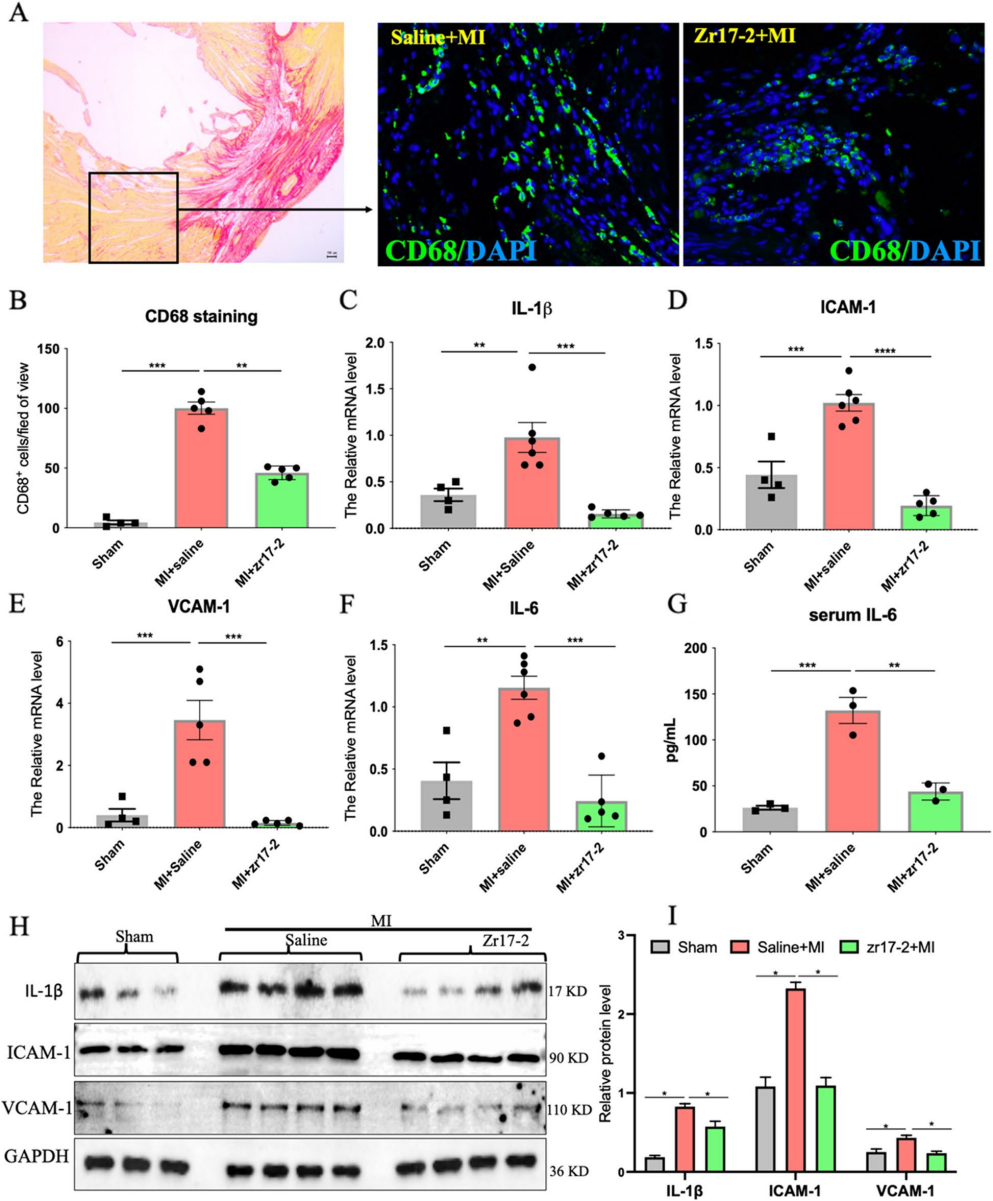
The effects of zr17-2 pretreatment on cardiac inflammation in MI rats. **A** Representative images of heart tissue stained with Sirius red or CD68 fluorescent antibody (green) and DAPI (blue) from the MI rats treated with zr17-2 or Saline. **B** Quantitative analysis of CD68 staining in the peri-infarction areas of the hearts. **C**-**F** The mRNA levels of inflammatory factors in the heart tissues such as IL-1β, VCAM-1, ICAM-1, and IL-6. **G** The serum level of IL-6. **H**-**I** Western blot analysis of the protein levels of IL-1β, ICAM-1, and VCAM-1 in the heart. β-actin was used as an internal control. IL-1β, interleukin 1 beta; ICAM-1, intercellular adhesion molecule 1; VCAM-1, vascular cell adhesion molecule 1; *n* = 3–6 in each group. ** *p* < 0.01, ***p < 0.001 and **** *p* < 0.0001

The inflammatory response to acute MI plays a critical role in determining MI size, and a persistent pro-inflammatory reaction can contribute to adverse post-MI cardiac remodeling [11]. We then evaluate the inflammatory response in the peri-infarction region of the post-MI rats. As shown in Fig. 2A, immunofluorescence staining of CD68, a marker of macrophage, showed that there existed a large amount of CD68^+^-macrophage infiltration in the peri-infarction region in the MI group, while this phenomenon was attenuated in zr17-2 treated group (Fig. 3A and B). Then the mRNA levels of inflammatory factors in the heart tissues were further determined. Interestingly, the mRNA levels of IL-1β, IL-6, VCAM-1, and ICAM-1 were all significantly increased in the MI group but were strikingly decreased by zr17-2 pretreatment (Fig. 3C F). In addition, we also detected the proinflammatory cytokine IL-6 protein level in the serum of MI rats and we found that the serum IL-6 level was significantly increased in MI rats, but almost resolved back to normal in zr17-2-pretreated rats (Fig. 3G). Consistently, the protein levels of IL-1β, ICAM-1, and VCAM-1 were also shown to be upregulated in the MI + saline group compared to that in the sham group but was resolved in the MI + zr17-2 group (Fig. 3H and I). Collectively, these results indicated an anti-inflammatory effect of zr17-2 in the context of the MI condition.

### zr17-2 pretreatment upregulated Nrf2 antioxidant genes in vivo and protected cardiac cells’ apoptosis from oxidative stress in vitro

**Fig. 4:**
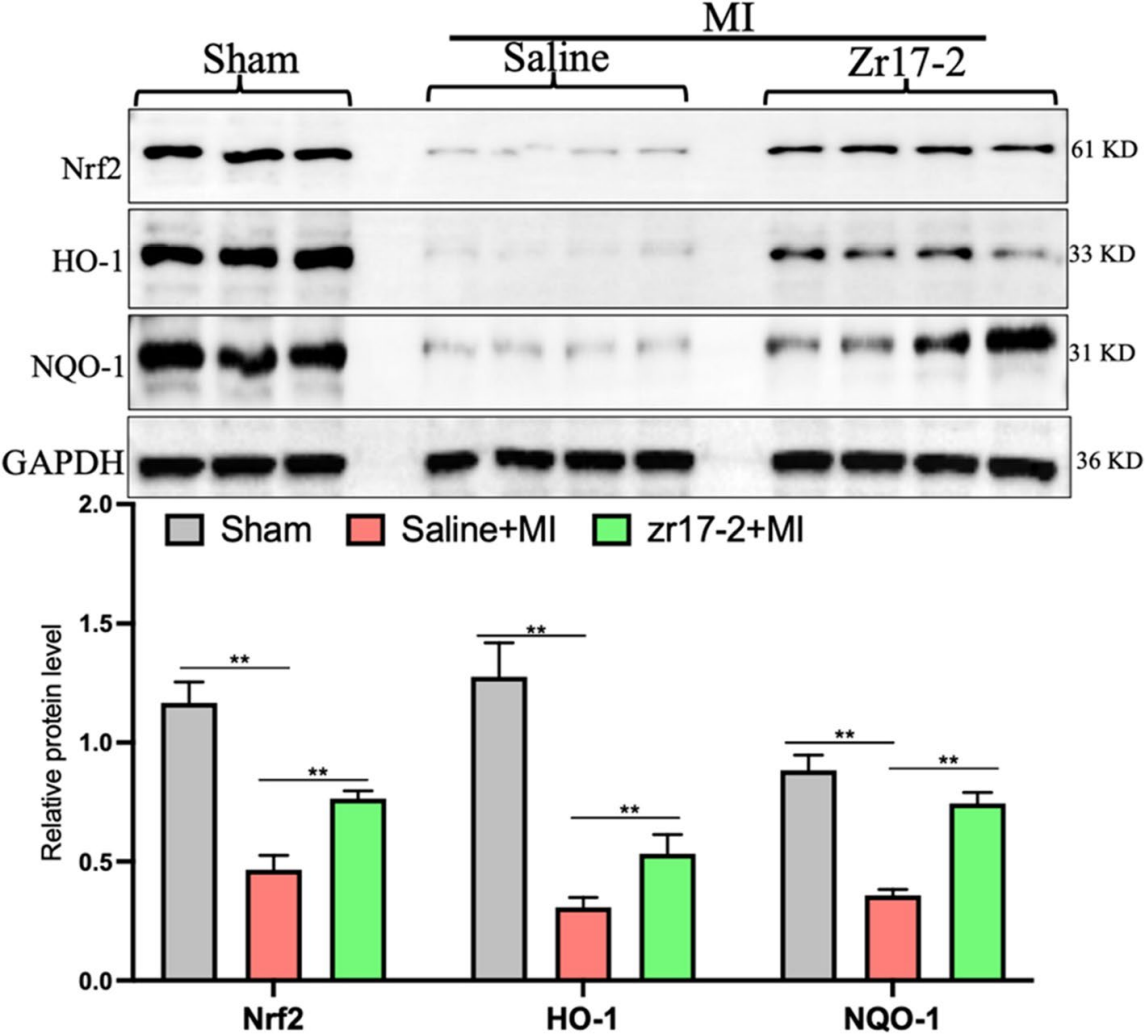
The effects of zr17-2 pretreatment on cardiac Nrf2 antioxidant genes in MI rats. Western blot analysis of the protein levels of Nrf2, HO-1, and NQO-1 in the heart tissues. Nrf2, nuclear factor erythroid 2-related factor 2; HO-1, heme oxygenase-1; NQO-1: NAD(P)H quinone dehydrogenase 1; *n* = 3–6 in each group. ** *p* < 0.01

Recently we discovered that CIRP is an upstream regulator of the Nrf2 antioxidant system in HEK293T cells and upregulation of CIRP could lead to increased Nrf2 antioxidant gene expression [12]. We then detected the expression of Nrf2 and its regulated downstream antioxidant genes such as HO-1 and NQO-1. Interestingly, the protein levels of Nrf2, HO-1, and NQO-1 showed significantly downregulated in the MI + saline group compared to that in the Sham group but were significantly recovered in the MI + zr17-2 group (Fig. 4). Collectively, these results suggested that upregulation of CIRP may increase cellular antioxidant capacity in the heart in the context of MI conditions.

**Fig. 5:**
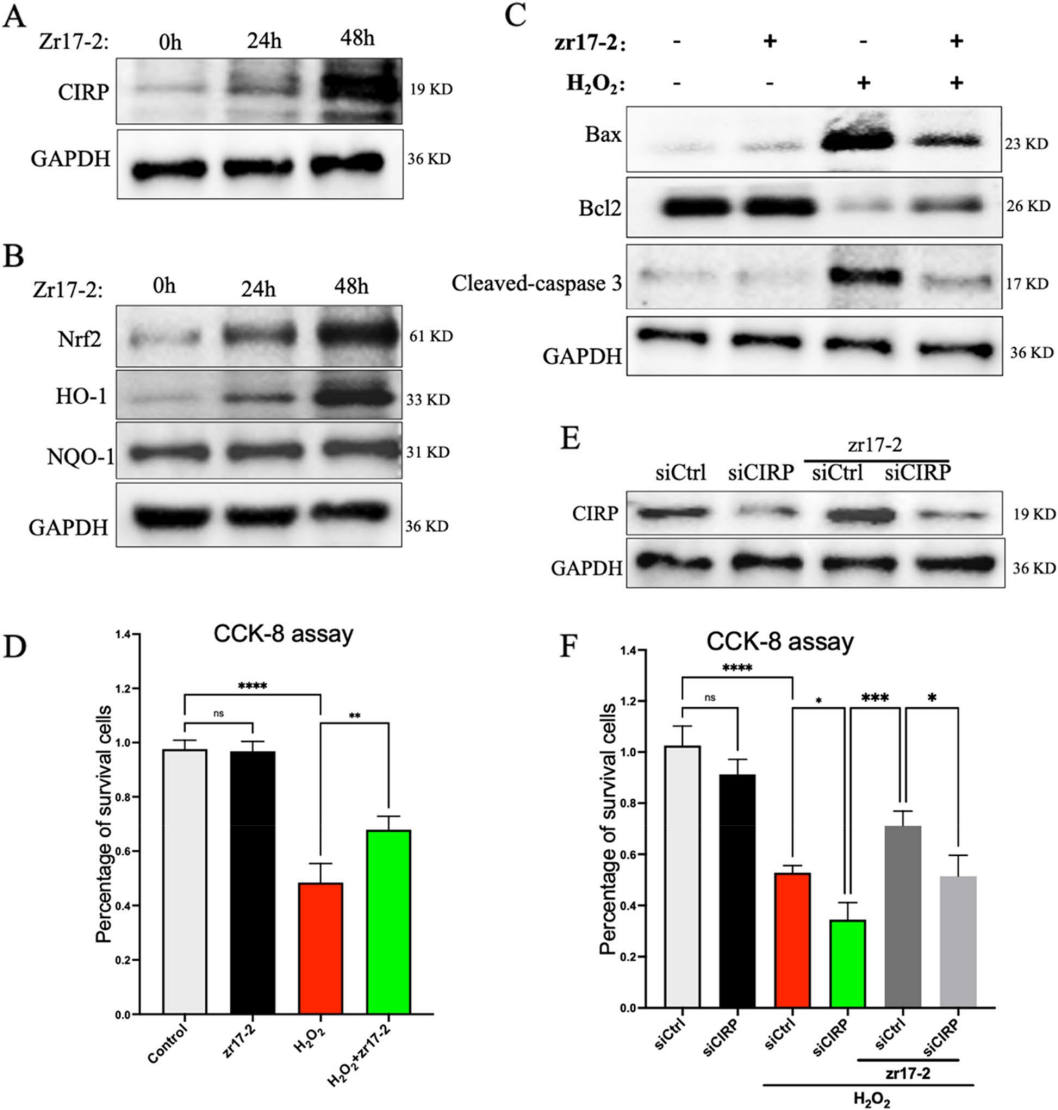
The effects of zr17-2 on cardiac apoptosis in hydrogen peroxide (H_2_O_2_)-stimulated H9C2 cells in vitro. **A** Western blot analysis of CIRP expression in response to zr17-2 treatment at different times. **B** Western blot analysis of Nrf2, HO-1, and NQO-1 in response to zr17-2 treatment at different times. **C** Western blot analysis of apoptosis-associated markers such as Bax, Bcl2, and cleaved-caspase 3 from H9C2 cells in response to H_2_O_2_ stimulation in the presence or absence of zr17-2 pretreatment. **D** Cell survival analysis of H9C2 cells by CCK8 assay in response to H_2_O_2_ stimulation in the presence or absence of zr17-2 pretreatment. **E** The effects of siRNA targeting the CIRP gene on the protein level of CIRP in vitro in the presence or absence of zr17-2. **F** Cell survival analysis of CIRP-deficient cells by CCK8 assay in response to H_2_O_2_ stimulation in the presence or absence of zr17-2 pretreatment. *n* ≥ 3 in each group. *p < 0.05, **p < 0.001 and ****p < 0.0001

As our in vivo studies demonstrated a protective role of zr17-2 in the MI heart, we then try to determine whether zr17-2 could directly elicit a cardioprotective effect in vitro cardiac cells in response to oxidative stress. We first evaluated the effect of zr17-2 on the protein level of CIRP in cardiac H9C2 cells. The dose of zr17-2 was chosen based on the previous work [9]. As shown in Fig. 5A, pretreatment with zr17-2 increased the protein level of CIRP in a time-dependent manner in H9C2 cells. In addition, we also tested the effect of zr17-2 on the protein levels of Nrf2, HO-1, and NQO-1 in cardiac cells. As shown in Fig. 5B, treatment with zr17-2 time-dependently elevated the protein level of Nrf2, HO-1, and NQQ-1. We then tested the cardioprotective effect of zr17-2 against oxidative injury in the hydrogen peroxide (H_2_O_2_)-stimulated H9C2 cell model. CCK8 assay showed that zr17-2 pretreatment attenuated the reduction in the viability of H9C2 cells stimulated with H_2_O_2_ (Fig. 5D). In addition, western blot analysis of the apoptotic proteins showed that both Bax and cleaved caspase-3 protein levels were significantly elevated in the H_2_O_2_-treated cells, whereas Bcl-2 levels were downregulated, compared to that in the control group (Fig. 5C). However, pretreatment with zr17-2 significantly decreased the protein levels of Bax and cleaved caspase-3 and increased the expression levels of Bcl-2 compared to those of the H_2_O_2_-treated cells (Fig. 5C). To further determine whether the cardioprotective effect of zr17-2 was mediated by increased CIRP, knockdown experiments were performed. siRNA targeting the CIRP gene was utilized and its gene-silencing effect was determined by western blot analysis in Fig. 5E. Then, CIRP-deficient cells or non-deficient cells were subjected to oxidative stress in the presence or absence of zr17-2, and we found that in the presence of zr17-2, silencing of CIRP lead to more cell death compared to that in CIRP non-deficient cells (Fig. 5F). Collectively, these results suggest a direct cardioprotective role of zr17-2 against oxidative injury in vitro and the important role of CIRP in mediating the cardioprotective effect of zr17-2.

## Discussion

Recent studies have suggested that CIRP exerted a protective effect against cell death under stress conditions in the hearts. For instance, in prolonged ex-vivo heart preservation under hypothermic conditions, CIRP KO hearts were shown to have more cell apoptosis and worse cardiac function, while CIRP transgenic hearts showed less apoptosis and better cardiac function [7, 13]. Mechanistic studies found that CIRP could positively and post-transcriptionally regulate the protein level of ubiquinone biosynthesis protein COQ9, an essential component in regulating the ubiquinone (CoQ10) biosynthesis, thus promoting ATP production and protecting cells against oxidative stress [7, 13, 14]. More interestingly, treatment with a CIRP agonist zr17-2 also extended heart preservation, coupled with enhanced expression of CIRP, increased CoQ10 and ATP levels, as well as promoted scavenging of reactive oxygen species [7, 13]. Taken together, these ex-vivo studies indicate a protective role of CIRP in the heart, and the application of CIRP agonist zr17-2 may have therapeutic potential in the heart under stress conditions. Our previous study showed that CIRP is downregulated in patients with heart failure and animal models of heart failure. However, the significance of such downregulation of CIRP in the development of heart failure is still unknown. In the present study, we used a CIRP agonist zr17-2 as a research tool to test the effects of elevating CIRP expression on the development of heart failure following acute MI. Interestingly, upregulation of CIRP by pretreatment with zr17-2 before the induction of the MI model significantly attenuated MI-induced cardiac dysfunction and dilation, coupled with reduced cardiac infarction size and improved cardiac remodeling. In addition, the beneficial effects of zr17-2 pretreatment on the heart were associated with the downregulation of inflammation and the upregulation of antioxidant genes in the heart. Taken together, our study suggests an important role of CIRP in the development of heart failure and demonstrated a beneficial effect of CIRP agonist zr17-2 in preventing the development of heart failure in the context of MI conditions, possibly via anti-inflammatory and anti-oxidant pathways.

More interestingly, we found an anti-inflammatory effect of CIRP agonist zr17-2 in vivo as evidenced by the reduction in the numbers of macrophage infiltration and the levels of inflammatory cytokine in the MI heart. Studies have shown that inflammatory response following acute MI plays a critical role in determining acute MI size and subsequent post-MI adverse LV remodeling [11]. Following acute MI, local cellular injury and death could initiate a pro-inflammatory response and result in the recruitment of inflammatory cells into the MI zone which may further induce the death of cardiomyocytes and extend ischemic injury beyond the original MI zone [11]. Therefore, anti-inflammatory therapy has the potential to reduce cardiomyocyte injury associated with MI. The identified anti-inflammatory role of zr17-2 may contribute to better cardiomyocyte preservation and subsequent cardiac function in the context of MI conditions. In addition, we also found that pretreatment with zr17-2 reduced IL-6 levels in serum in the MI rats. As clinical studies have provided evidence that targeting serum IL-6 with an anti-IL-6R antibody could reduce peri-procedural myocardial injury in acute MI patients [15], the effect of zr17-2 on serum IL-6 level may also contribute to less cardiac injury.

Nrf2 regulates the transcription of antioxidant defense enzymes in the cell and is important for regulating cellular resistance to oxidative stress. Activation of antioxidant genes, including HO-1 and NQO-1, is mediated by Nrf2, which translocates into the nucleus and binds to the antioxidant response element in the DNA promoter region. Nrf2 and its regulated downstream antioxidant genes have been reported to be downregulated in post-MI hearts, contributing to the occurrence of an oxidative stress injury in the MI hearts and the development of heart failure [16–18]. In our study, we also observed that the gene levels of Nrf2, HO-1 and NQO-1 were markedly downregulated in the post-MI hearts. However, the reduction in the expression of Nrf2 and its downstream antioxidant genes were significantly recovered in the zr17-2-pretreated group, suggesting a role of zr17-2 in regulating cellular antioxidant capacity. In addition, we also evaluated the direct effect of zr17-2 on the expression of Nrf2 and its downstream antioxidant genes in the heart in vitro and in vivo. Interestingly, the protein levels of Nrf2, HO-1, and NQO-1 could also be elevated by the direct treatment with zr17-2 both in vitro and in vivo (Fig. 5B and supplemental Figure S1). Moreover, our in vitro study also demonstrated a direct cardioprotective effect of zr17-2 against H_2_O_2_ stimulation. These results suggest an antioxidant effect of zr17-2 in the heart. Importantly, genetically knocking down CIRP could block the protective effect of zr17-2 against oxidative stress in response to H_2_O_2_ stimulation. These results suggests that the antioxidant role of zr17-2 is dependent on CIRP. Our previous study also showed that CIRP can protect cells against oxidative stress injury, as silencing of CIRP leads to more cell apoptosis in response to H_2_O_2_ stimulation [8]. Furthermore, CIRP overexpression in neural cells has been shown to increase key antioxidant enzyme levels, including glutathione (GPx), superoxide dismutase (SOD), and catalase (CAT) [19]. Interestingly, the antioxidant enzymes listed above can be controlled by Nrf2 [20]. Moreover, our recent mechanistic study also identified the Nrf2 pathway as the downstream target of CIRP, and CIRP knockdown could lead to the downregulation of Nrf2 and its regulated antioxidant genes [12]. Collectively, these results indicate a possible role of CIRP and its agonist zr17-2 in regulating the Nrf2 antioxidant system, and further studies in vitro cultured primary cardiomyocytes are needed to verify the relationship between CIRP or its agonist zr17-2 in regulating the Nrf2 antioxidant system.

It should be noted that the possible toxicity of zr17-2 was not evaluated in our present study. As it is of vital importance to determine the safety of drugs, further toxicity studies should be performed to evaluate the possible effects of zr17-2 on other organs such as the liver and kidney. In addition, we did not evaluate the systemic hemodynamic parameters, which would be a good complement to the echocardiography analysis and would make the conclusion more convincing. In the present study, we only evaluate the early effects of zr17-2 on the post-MI hearts at 7 days, the evaluation of the cardiac function and histology at later time points including 2 or 4 weeks after MI would be more valuable to determine the long-term effects of zr17-2 on the heart. Another limitation is that a drug control group was lacking in vivo. Whether zr17-2 has any direct effects and inflammatory response is still unknown and further studies are needed to determine this. Additionally, CIRP has also been considered a proto-oncogene that activates several cell cycle-related proteins leading to cancer promotion. Therefore, whether elevation of CIRP by zr17-2 has any pro-carciogenic effects needs to be investigated. In the present study, as zr17-2 was only administered for a short time (three injections in one week), we did not observed any apparent abnormalities in the outward appearance of the skin, lungs and live of the experimental rats. Thus, longer-term observational studies are warranted to determine the long-term effects of zr17-2 administration.

## Conclusion

Taken together, our study demonstrated a beneficial effect of CIRP agonist zr17-2 in preventing the development of heart failure in the context of MI conditions possibly via ani-inflammatory and anti-oxidant pathways.

### Supplementary Information

**Supplementary Material 1.**

## Data Availability

The datasets used and analyzed during the current study are available from the corresponding author upon reasonable request.
