# Supplementary material for: Upregulation of CIRP by its agonist prevents the development of heart failure in myocardial infarction rats
Source: BMC Cardiovasc Disord. 2024 Mar 27;24:185. doi: 10.1186/s12872-024-03852-9 (PMC10967100; doi:10.1186/s12872-024-03852-9)
Supplement: Supplementary file 1 — Supplementary Material 1. [file 12872_2024_3852_MOESM1_ESM.pdf]

Supplemental Figure S1

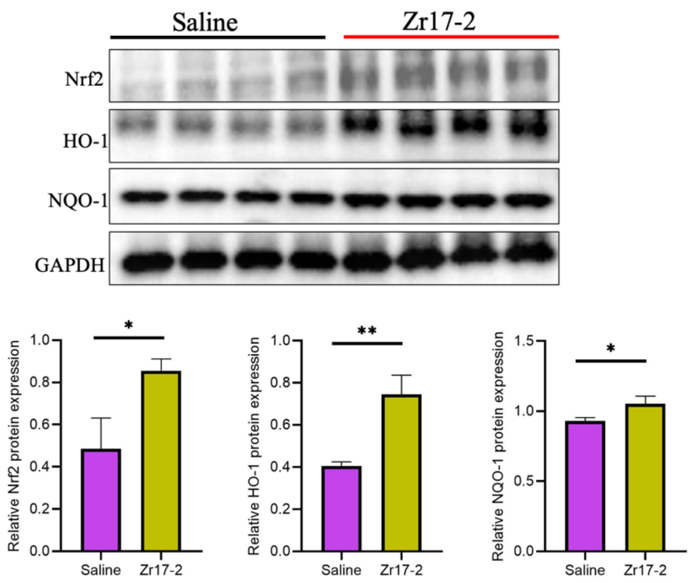

Supplemental Figure S1: Effects of zr17-2 on the expression of Nrf2, HO-1 and NQO-1 in normal SD rats. The SD rats was treated with zr17-2 (20nmol/kg, i.p.) or saline once every other day 3 times, followed by the evaluation of cardiac genes expression by western blot. *p<0.05, **p<0.001.

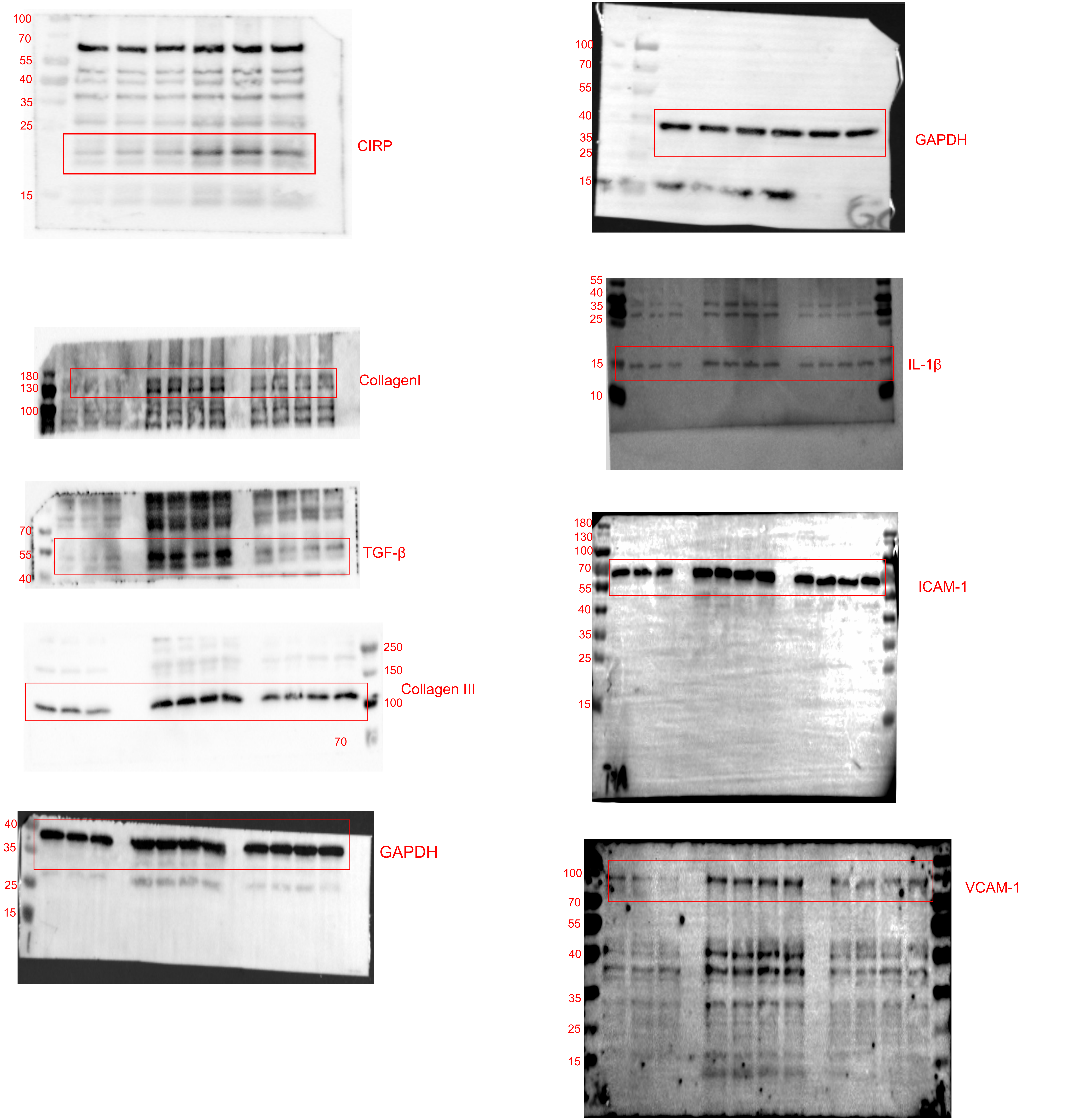

100
70
55
40
35
25
15
CIRP
100
70
55
40
35
25
15
GAPDH
180
130
100
CollagenI
55
40
35
25
15
10
IL-1β
70
55
40
TGF-β
180
130
100
70
55
40
35
25
15
ICAM-1
250
150
100
70
Collagen III
40
35
25
15
GAPDH
100
70
55
40
35
25
15
VCAM-1

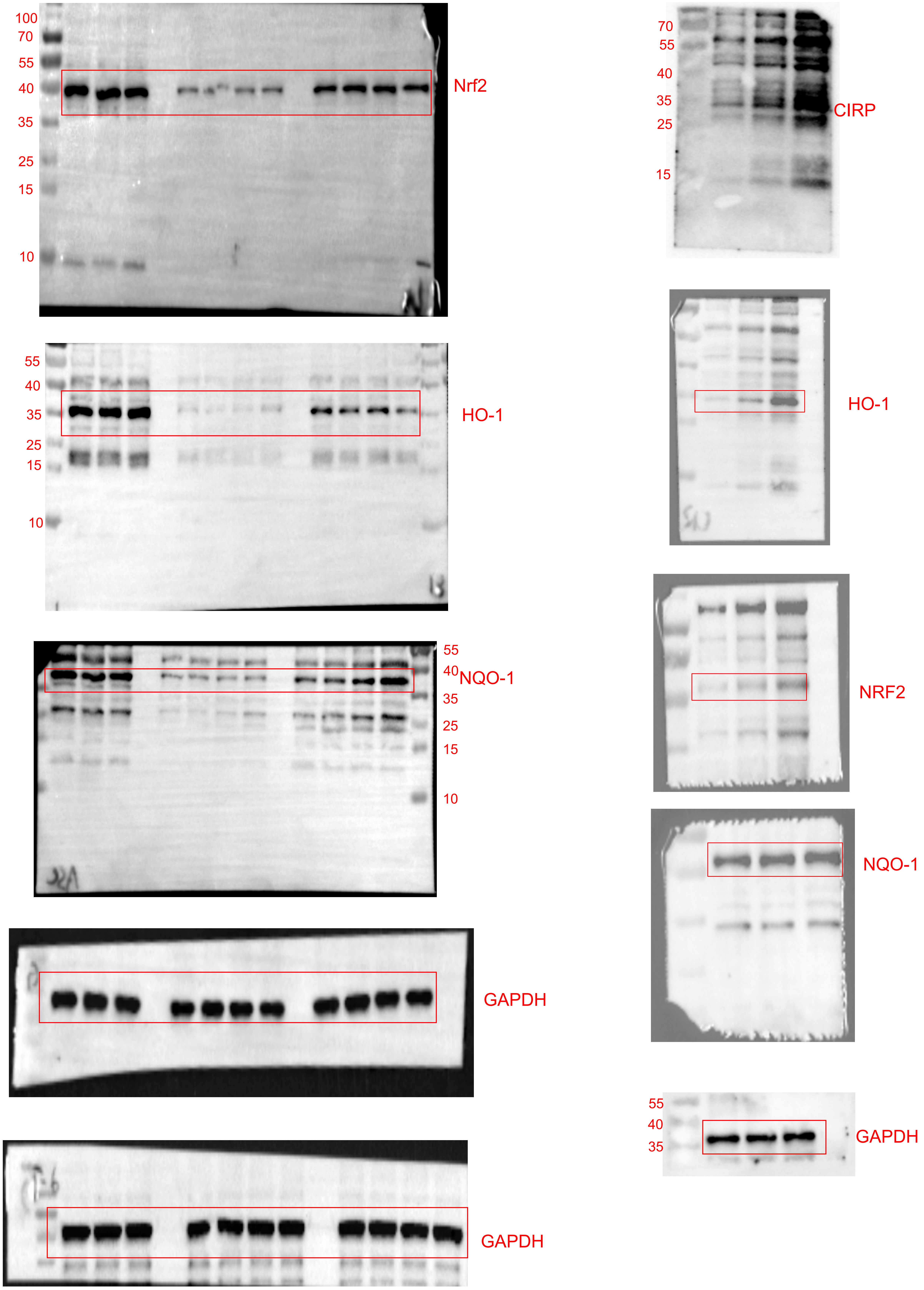
100
70
55
40
35
25
15
10
Nrf2
55
40
35
25
15
10
HO-1
55
40
35
25
15
10
NQO-1
GAPDH
GAPDH
70
55
40
35
25
15
CIRP
HO-1
NRF2
NQO-1
55
40
35
GAPDH

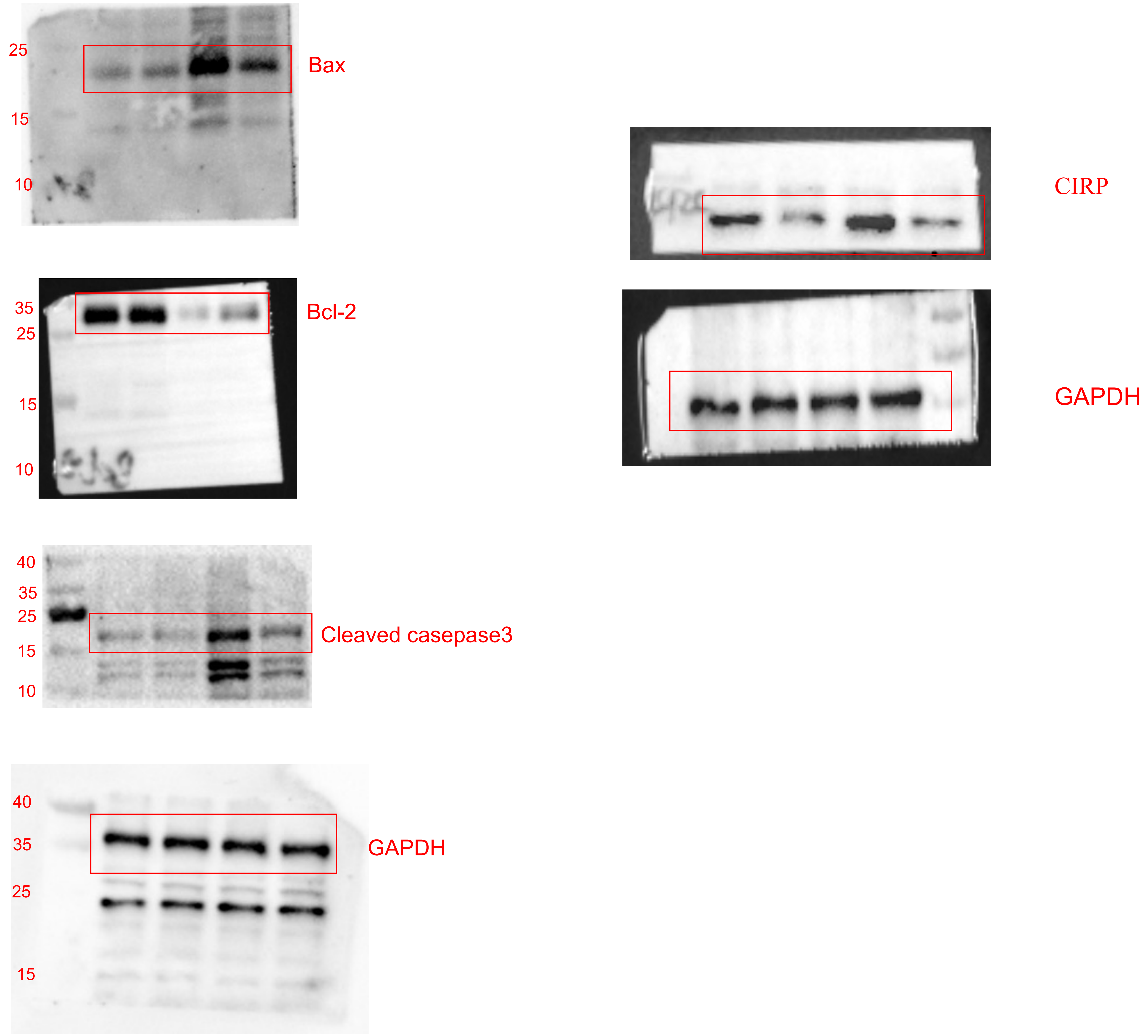
25
15
10
Bax
35
25
15
10
Bcl-2
40
35
25
15
10
Cleaved casepase3
40
35
25
15
GAPDH
CIRP
GAPDH
